# Urban networks among Chinese cities along "the Belt and Road": A case of web search activity in cyberspace

**DOI:** 10.1371/journal.pone.0188868

**Published:** 2017-12-04

**Authors:** Lu Zhang, Hongru Du, Yannan Zhao, Rongwei Wu, Xiaolei Zhang

**Affiliations:** 1 State Key Laboratory of Desert and Oasis Ecology, Xinjiang Institute of Ecology and Geography, Chinese Academy of Sciences, Urumqi, China; 2 Department of Geography, Ghent University, Gent, Belgium; 3 University of Chinese Academy of Sciences, Beijing, China; 4 Sino-Belgian Joint Laboratory of Geo-information, Urumqi, China; 5 Sino-Belgian Joint Laboratory of Geoinformation, Gent, Belgium; 6 Institute of Geographic Sciences and Natural Resources Research, Chinese Academy of Sciences, Chaoyang District, Beijing, China; Beihang University, CHINA

## Abstract

“The Belt and Road” initiative has been expected to facilitate interactions among numerous city centers. This initiative would generate a number of centers, both economic and political, which would facilitate greater interaction. To explore how information flows are merged and the specific opportunities that may be offered, Chinese cities along “the Belt and Road” are selected for a case study. Furthermore, urban networks in cyberspace have been characterized by their infrastructure orientation, which implies that there is a relative dearth of studies focusing on the investigation of urban hierarchies by capturing information flows between Chinese cities along “the Belt and Road”. This paper employs Baidu, the main web search engine in China, to examine urban hierarchies. The results show that urban networks become more balanced, shifting from a polycentric to a homogenized pattern. Furthermore, cities in networks tend to have both a hierarchical system and a spatial concentration primarily in regions such as Beijing-Tianjin-Hebei, Yangtze River Delta and the Pearl River Delta region. Urban hierarchy based on web search activity does not follow the existing hierarchical system based on geospatial and economic development in all cases. Moreover, urban networks, under the framework of “the Belt and Road”, show several significant corridors and more opportunities for more cities, particularly western cities. Furthermore, factors that may influence web search activity are explored. The results show that web search activity is significantly influenced by the economic gap, geographical proximity and administrative rank of the city.

## Introduction

The initiative of jointly building the “Silk Road Economic Belt and the 21st Century Maritime Silk Road” (hereafter referred to as “the Belt and Road”) was proposed by Chinese President Xi Jinping in 2013. The initiative aims to establish a new pattern of trade and an infrastructure network that connects Asia, Europe and Africa [1, 2]. In addition to trading cooperation and the economic situation, cultural exchanges and investment policy [2] will be covered. In this case, the initiative will generate numerous city centers, which may be defined as nodes to facilitate interactions between major centers [3, 4]. Since the initiative was proposed, for example, Changchun attempted to build a white science and technology park to focus on the development of an international cooperation platform for science and technology. Shenzhen set up an international museum aimed at the integration of the culture and technology of “the Belt and Road”; moreover, additional free trade zones have been established in Xi’an, Shenzhen and Urumqi (see https://www.yidaiyilu.gov.cn/info/iList.jsp?cat_id=10004). The centrality of these cities has been strengthened; as a result of both policy and attention, they have become popular cities.

A city’s position determines its benefit from the initiative. More specifically, a city’s power arises from the forms of interaction and exchange that occur through complex networks [5]. Among these networks, the co-present interaction shrinks the space and time between each other and constructs more closely integrated ties and relationships [6]. According to Castells’s argument, physical proximity and face-to face interaction were emphasized in an industrial society, whereas the diffusion of information and communication have been emphasized in the information age [7]. Additional evidence has indicated that organizations and businesses rely on high-quality data communications and low costs for their telecommunications needs [8, 9].

In this paper, we have three central aims. The first aim introduces the methodological framework and argument that information flow is as an appropriate database for examining the increasing importance of knowledge opportunities for cities. Second, the informational ranking of Chinese cities along “the Belt and Road” is derived. The paper uses the Baidu search volume in a comparative analysis of an informational approach, and Chinese cities along “the Belt and Road” are selected for a case study to determine how these cities are merged in cyberspace and the specific opportunities that are offered in the context of “the Belt and Road”. Third, the research field is extended by examining the factors that affect information flow.

The remainder of this paper consists of four main sections. The first section discusses the theoretical rationale for an analysis of urban networks among Chinese cities along “the Belt and Road”. The second section briefly describes the selected cities, calculation method and hypotheses for the potential influencing factors. The results are presented in the third section. The fourth section discusses the main findings and potential drawbacks of the data outlined in this section. Following this section, an overview of potential avenues for further research is presented.

The next section discusses the theoretical rationale for an analysis of urban networks among Chinese cities along “the Belt and Road”. We subsequently discuss the debate regarding “the Belt and Road” to provide context and outline its remit. Following that, we acknowledge that exploring urban hierarchies among Chinese cities along “the Belt and Road” is essential to advance our understanding of center cities and their dynamic connections. Then, we argue the conceptualization of hierarchies and networks. We acknowledge that a city’s network of information flows presents an amalgam of hierarchical and networked characteristics. Given this understanding, we subsequently introduce and discuss in detail the feasibility of using the Baidu index as a source of information flows.

## “The Belt and Road”, urban hierarchies and information flows

### “The Belt and Road”: Context

Cities may be connected at different scales. Empirical studies tend to analyze the spatial organization by examining functional linkages at different scales, including global [10–13], regional [14, 15] and national [16–18]. However, many economic communities have experienced greater functional linkage because of more investments within the region. Furthermore, the spatial paradigms that are inherent to these regional initiatives indicate the reproduction of capitalist developmental ideas expressed particularly in the form of networks, which thus become a feature of the contemporary global political economy [19].

“The Belt and Road” initiative is a “spatial fix” [19] expected to facilitate interactions among numerous city centers. Certainly, the initiative of “the Belt and Road” would generate a number of centers, both economic and political, that would facilitate more interaction among them. Research on cities in this area would not only help guide the long-term development of “the Belt and Road” but would also make economic centers more attractive by providing productivity benefits.

### Urban hierarchies

In Castells’s seminal work, he argues a network society encompasses a “global network” of cities rather than a few urban cores at the top of the hierarchy [7]. Urban research has subsequently undergone a shift from the hierarchical arrangement of city-entities to a networked ontology. More specifically, a hierarchy implies a ranking of cities with more-or-less power, whereas a network suggests a flattened space with mutuality and cooperation [20]. As cities have been locked into uneven power networks, emphasizing cooperation rather than competition within urban hierarchies seems reasonable [21]. The rise of information and communication technologies (ICTs) has complicated the relationship between urban places and digital information. Thus, in an information age, the main task for a city is to produce and use informational data. Certain cities always have more advantages than others in terms of the production and consumption of discourse on economic effect, particularly with respect to policy. Therefore, the network ontology should accommodate cyberspace-based city rankings [22]. Examining cities from the perspective of their urban rank makes sense, although the discussion of an urban hierarchy may be unfashionable.

The use of inter-city connection data has become an increasingly popular way to examine urban hierarchies [23]. This paper employs a network approach to explore urban hierarchies among Chinese cities along “the Belt and Road”. Employing the network approach is also consistent with the new network paradigm presented in the “urban system” literature.

### Information flows: A data source

In the present “information age”, the quickening and deepening connections among cities, which are considered characteristic of the contemporary globalizing world, call for novel ways of understanding the relative importance of cities [24]. The rapid development of ICTs has led to the reshaping of urban society and economies, for example, by changing information exchange, communication, and the links between cities [7, 25]. Cyberspace is a virtual geography created by ICTs [26], in which the internet is a remarkable fusion of information types, media, and operators [27], and more flows of people, material and information, for example, are generated. The increasing use of the internet and its applications is also perceptible in interrelationships between cities [28], and the internet has various spatial structures [29, 30]. Scholars have simultaneously questioned the relevance of the internet to the original urban hierarchy and determined that the exchange of information among cities would lead to a more complex urban hierarchy and not simply follow the existing urban network structure [31–33].

It is a truism of the information age that information is everywhere, however, with respect to information relevant to scholars, the identification and utilization of “good” data are relatively new developments [22]. Moreover, empirical studies tend to present two specific problems: (1) Many studies have shown that there is an infrastructure-orientated focus [34, 35]. Of these studies, the structure of links among IP addresses [36], internet backbones [37, 38] and tracing information “itself” have been used to portray the internet networks that have been the subject of network analysis. However, the infrastructure-orientated focused data cannot present the connectivity between two cities. The fact that a city has a high degree of infrastructure is not an indication that it connects well with other cities [39]. (2)Telecommunications are, to some extent, invisible compared with visible indicators, such as airports [40, 41]. Therefore, in internet studies, only a small amount of comparable data are available to accurately measure information flows [39, 42, 43]. This is described as “the dirty little secret of cities research” [44].

Therefore, informational infrastructures and associated material flows between cities must be investigated, as well as the evolving cyberspaces of cities in relation to digital information [22]. The content-based analysis of cyberspace has been used to trace out dyadic interconnections between cities by obtaining counts of web pages [28, 45]. A novel heuristic research method from a web search engine has proven to be appropriate for measuring urban connectivity in information networks [22].

### Deriving inter-city connections from the Baidu index

The internet may be considered a window into a city. Web search activity has become an important way for individuals to learn about a city, and it is regarded as the informational link [46, 47]. According to the "39^th^ China Internet Development Report", by the end of 2016, the number of Chinese internet users reached 731 million [48]. Because of the national government’s policy of internet censorship [49], Baidu is the main web search engine in China instead of Google web search. With more than 665 million active users accounting for 91% of the total (see http://news.xinhuanet.com/fortune/2017-02/24/c_129495316.htm), Baidu has become the most widely used web search engine in China.

To understand the type of web search activity among cities, six cities from the study area are selected, and the nationwide search contents for these cities are sorted (Table 1). The large search content is recorded according to baidu.com using a combination of the city name and the search content. For example, the combination of “Beijing” and “weather” had the largest search volume when individuals nationwide searched for Beijing. The most common combination for the selected cities was weather. The large search content also includes Yaohao, subway, university, and coach when people nationwide searched for Beijing. When individuals searched for Shanghai, the predominant search content included Disney, university, subway and traffic. For the famous tourist city Sanya, the largest search content included photograph, tenement, wedding veil and Haikou. Most of these search contents imply strong connections among cities. For example, when individuals in Beijing search for weather, subway and other transportation routes into Shanghai, they tend to have the following two reasons: (1) they would go to Shanghai in the following days, or (2) they have family members or friends in Shanghai. In this case, some aimless search volume that is also included is assumed to be temporal. The previously described search content listed showed a strong desire for connections (e.g., business, leisure, residence, or employment) behind these web search activities.

**Table 1:** The ranking of search content for cities in one week (from 2016.12.26 to 2017.01.01).

Ranking of search content	Beijing	Shanghai	Chongqing	Shantou	Lhasa	Sanya
**1**	Weather	Weather	Shishicai ^a^	Weather	Weather	Weather
**2**	Yaohao ^a^	Disney	University	University	Tibetan	Photograph
**3**	Subway	University	Weather	Haitong ^a^	Train	Tenement
**4**	University	Subway	Occupation	Recruit	Chengdu	Wedding veil
**5**	Coach	Traffic	Travel	Stock	Beijing	Haikou

^a^ Yaohao grants car registrations via a lottery system. Shishicai is a type of welfare lottery issued by the China welfare lottery distribution management center, and underwritten by the Chongqing welfare lottery distribution center. Haitong is a securities joint stock limited company in China.

When analyzing the Baidu web search, most of the search content may be traced back to the connection among cities. Specifically, the flow is expressed as information flow, however, in essence, it is an integrated flow including people, material and information. Interestingly, Baidu web search activity provides the possibility for a city-based analysis based on users’ geo-tagged places, which typically correspond to their location. Baidu is a web search engine for data sharing based on the massive behaviors of internet users. The Baidu index may provide researchers with the scale of a keyword, its evolution over time, and the distribution of internet users who search for these words. For example, when a user in Shanghai searches for a combination of content that includes the key word “Beijing”, the search volume referred to as the Baidu index would be recorded. Key words such as “Beijing road” are not included. Specifically, when users in Beijing are searching for combinational contents including the key word “Shanghai”, subtitled Disney, or university, these web search data are subsequently stored in the Baidu index. Therefore, the connection between Shanghai and Beijing, which is bidirectional, may be calculated based on the web search activity of users.

In contrast to infrastructure-orientated approaches, in which information flows are measured, the Baidu index approach deals with virtual flows. The Baidu index is operated standardly based on its web search data that may present not only information infrastructures but also their associated material flows between cities, which indicates an overview of urban networks among the cities.

Against this background, the exploration of urban hierarchy among Chinese cities along “the Belt and Road” has two meanings. First, this work presents a way to capture connections and identities of places using associated material flows in China, which has the largest number of web search engine users worldwide. Second, this study mapping urban networks among Chinese cities along “the Belt and Road” also has substantial realistic significance, particularly for understanding the center city along “the Belt and Road”.

## Area, methods and hypothesis

### Selection of cities

Chinese cities along “the Belt and Road” have been introduced as a trial to examine how information flows may be used as a framework to empirically study the contemporary global political economy. On March 28, 2015, the National Development and Reform Commission, Ministry of Foreign Affairs, and Ministry of Commerce jointly issued the “Vision and Actions on Jointly Building Silk Road Economic Belt and 21st-Century Maritime Silk Road”[50]. In the report, 18 provinces in China are identified as key provinces, including 6 northwestern, 3 northeastern, 3 southwestern, 5 coastal, and 1 inland province. Moreover, 18 node cities in China are identified, including Beijing, Chengdu, Zhengzhou, Wuhan, Changsha, Nanchang, Hefei, Tianjin, Ningbo, Shenzhen, Zhanjiang, Shantou, Qingdao, Yantai, Dalian, Xiamen, Quanzhou and Sanya. These cities are introduced as the main connector span of China with other countries in the initiative of “the Belt and Road”. Therefore, 18 provincial capitals and 18 node cities are selected for analysis in this paper (Table 2).

**Table 2:** The 36 selected Chinese cities along “the Belt and Road”.

City	Rank of GDP per person	Rank of population	City	Rank of GDP per person	Rank of population
Chongqing	12	1	Beijing	2	3
Shanghai	1	2	Chengdu	7	5
Fuzhou	21	19	Zhengzhou	14	10
Guangzhou	3	6	Wuhan	6	8
Hangzhou	8	12	Changsha	10	20
Haikou	33	33	Nanchang	23	27
Nanning	26	23	Hefei	20	16
Kunming	24	24	Tianjin	5	4
Lhasa	36	36	Ningbo	11	17
Harbin	19	9	Shenzhen	4	7
Changchun	22	18	Zhanjiang	29	21
Shenyang	15	15	Shantou	31	26
Hohhot	27	31	Qingdao	9	11
Xining	34	32	Yantai	16	22
Yinchuan	32	34	Dalian	13	25
Lanzhou	30	29	Xiamen	25	28
Xi’an	18	13	Quanzhou	17	14
Urumqi	28	30	Sanya	35	35

Sources: All data are obtained from the <Urban statistical yearbook of China 2016>

Chinese cities along “the Belt and Road” were selected for a case study to investigate how these cities are merged in cyberspace and the specific opportunities that are offered in the context of “the Belt and Road”. The results of this study indicated which of the 36 cities are nearly in intensive collaboration and which cities are removed from this type of collaboration. This research may help the Chinese government provide relatively preferential policies to these node cities in the future.

### Data calculation based on the Baidu index

#### Construction of the inter-city informational connection matrix

Given that web search activity produced between two cities may be explored via https://index.baidu.com/, and the web search volumes may be represented by the Baidu index between two cities, an inter-city informational connection matrix may be constructed with data from the Baidu search engine. There are 1260 links among the 36 cities each year, thus, three matrices spanning the years 2011, 2014, and 2016 are built. All collected data comply with the terms of service for the Baidu website.

#### Estimation of the index of urban network characteristics

The data process in this paper includes four steps.

① Standardizing the connectional data among 36 cities. The specific steps are as follows:
  $$X_{ij}^{\prime }=\frac{X_{ij}}{\sum_{j=1}^{n}X_{ij}}$$
  where n represents the number of research cities; X_ij_ represents the web search volume of city i, in connection with city j; $\sum_{j=1}^{n}X_{ij}$ represents the web search volume of city i to other cities; and $X_{ij}^{\prime }$ presents the standardized data.
② Based on the first step, the second step is to calculate the city’s external connection index. The external connection index reflects the urban hierarchy in the network system.
  $$Y_{i}=\sum_{j=1}^{n}X_{ij}^{\prime }-X_{ii}^{\prime }$$
  where Y_i_ represents the city’s external connection index which reflects the city’s external contact strength in the entire network system; $X_{ij}^{\prime }$ is the standardized web search volume of city i to city j; and $X_{ii}^{\prime }$ is the standardized web search volume of city i to itself.
③ The third step is to calculate the connection degree between cities based on the first step. The indicator characterizes the informational contacting degree among cities in urban networks. The calculation is as follows.
  $$Z_{ij}=X_{ij}^{\prime }*X_{ji}^{\prime }$$
  where Z_ij_ represents the connection degree between cities i and j, $X_{ij}^{\prime }$ is the standardized web search volume of city j to city i, and $X_{ii}^{\prime }$ is the standardized web search volume of city i to itself.
④ The fourth step is to calculate the connection degree of each city for characterizing each city’s connection to other cities within the urban network system. The specific calculation is as follows.
  $$W_{i}=\sum_{j=1}^{n}Z_{ij}-Z_{ii}$$
  where W_i_ represents the connection degree of city i, Z_ij_ represents the connection degree between cities i and j, and Z_ii_ represents the connection degree between cities i and i.

#### Construction of the inter-city spatial distance matrix for all 36 cities

Euclidean distances between 36 cities are calculated, and a spatial distance matrix is established. The research considers the significant changes that have occurred in inter-city transportation distances over the previous 20 years as well as the vast territory of China. This paper examines only straight-line distances; in the 36×36 matrix, the minimum distance is 74 km from Xiamen to Quanzhou, and the maximum distance is 3504 km from Urumqi to Sanya.

### Hypotheses of underlying factors that affect web search activity

Factors such as city size (in demographic or economic terms), policies, physical distance and borders may affect the spatial interactions between cities [51]. From the perspective of web search activity, the question arises as to whether the urban hierarchy will continue to be affected by the economic, geographical and administrative factors? We subsequently formulate a hypothesis regarding how the underlying factors affect web search activities according to empirical studies.

(1) Assumption 1: cities with high levels of economic development tend to have high web search activity.
  The presence of information cyberspace often depends more on informational infrastructure [52]. In 1977, the viewpoint that the network of an urban system tends to spread among economically developed cities was proposed by Pred [53]. Therefore, this paper assumes that a city with a high level of economic development will tend to have a high web search activity.
(2) Assumption 2: web search activity tends to occur between cities with large economic gaps.
  We define the variable A_ij_ as the economic gap to show the extent of the economic gap between two cities. In this paper, GDP per capita is chosen to minimize the impact of the population and GDP. The economic gap between two cities is calculated as
  $$A_{ij}=\frac{1}{\left(|G_{i}-G_{j}|\right)}$$
  where i and j represent cities i and j (i≠j), respectively, G_*i*_ represents the GDP per capita of city i, and *G*_*j*_ represents the GDP per capita of city j. A smaller A_ij_ indicates a larger economic gap between cities i and j whereas a larger A_ij_ indicates a smaller the economic gap between cities i and j.
  This article assumes that web search activity tends to occur between cities with a large economic gap.
(3) Assumption 3: high administrative rank cities tend to have more opportunities to gain high web search activity.
  The influence of local government is mainly reflected in the intervention of the regional economic pattern, which is referred to as the administrative region economy [54]. High administrative rank cities tend to be more attractive for their information and policy advantages [55]. Therefore, this paper assumes that high administrative rank cities tend to have more opportunities and higher web search activity among cities.
(4) Assumption 4: web search activity is affected by geographical proximity.
  Urban network theory suggests that links among cities are not equivalent. Moreover, a rule of distance attenuation exists [56]. Therefore, this paper assumes that web search activity is affected by geographical proximity.

## Results

### Analysis of inter-city connections

#### Characteristics of web search activity and its evolution

**Fig 1:**
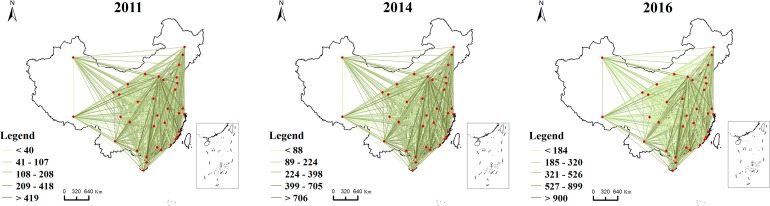
Evolution of web search activity among 36cities along “the Belt and Road” from 2011to 2016.

Combined with the city location, informational connection matrices of 2011, 2014 and 2016 were used to investigate the spatial characteristics of the network evolution (Fig 1). Overall, for 2011, 2014 and 2016, the web search activity significantly increased among cities along “the Belt and Road”. More specifically, in 2011, the total Baidu index was 115,845, and its value increased to 263,583 in 2014. Moreover, compared with the value in 2011, the value increased by 1.66 times in 2016, reaching 308,687. The average annual growth rate in inter-city informational connections was 166% among the selected years, which suggests a rapid growth trend.

Urban networks have gradually become more balanced. Among all cities, informational connections among 18 cities account for 68% of the total, and the information connection of Beijing, Guangzhou, Shenzhen, and Shanghai in 2011 accounts for 19.7% of the total. Specifically, we identify 22,843 links among four cities (Beijing, Guangzhou, Shenzhen, and Shanghai) in 2011, 47,643 in 2014, and 51,034 in 2016, which account for 19.7%, 18.9%, and 18.5%, respectively, of the total connections among the selected cities. Furthermore, a polycentric pattern is explicitly present in 2011, and a polycentric structure is maintained in Beijing and Shanghai in 2014, whereas a homogenized structure develops prior to 2016.

**Fig 2:**
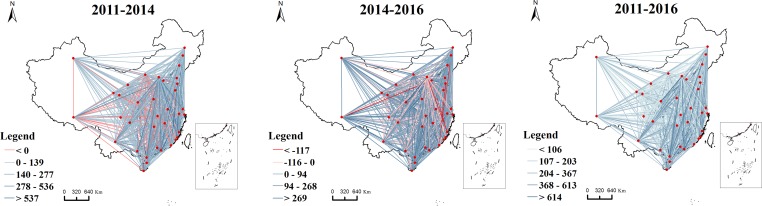
Changes in web search activity among cities in different years.

The network has undergone continuous adjustment, in which web search activity experienced a process of weakening between certain cities and strengthening between other cities. Compared with 2011, links among Hohhot, Lhasa and other cities significantly weakened in 2014. In contrast, links among Beijing, Shanghai, Guangzhou and other cities have significantly strengthened, which led to the central role of these cities. During 2014 and 2016, the links among Beijing, Kunming and other cities significantly weakened, resulting in a relatively homogeneous structure across the entire network in 2016. Although the links among cities have been reduced (or enhanced) in the previous two years, the links in 2016 have increased to varying degrees compared with those in 2011 (Fig 2).

#### Spatial distribution characteristics of web search activity

The web search process between cities is bidirectional, indicating that the web search activity between cities includes two aspects. For example, web search activities of city i on other cities and other cities on city i are both included. In this paper, two definitions are provided as follows: the sending Baidu index is the total web search volume of city i for other cities, and the receiving Baidu index is the total web search volume of other cities for city i.

**Fig 3:**
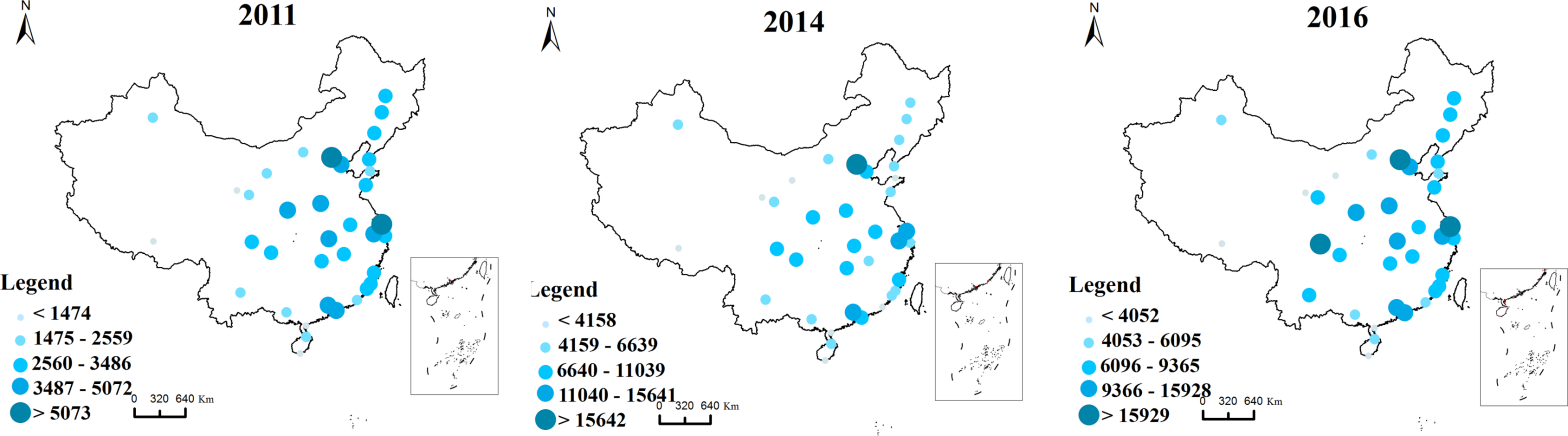
The sending Baidu index among “the Belt and Road” cities.

Using the method of natural breaks, an apparent pyramidal hierarchy is present as indicated in Fig 3, which shows that Beijing and Shanghai are the central cities, and Guangzhou, Shenzhen, Tianjin, Zhengzhou and Wuhan are the sub-central cities in 2011. During 2014, the sending Baidu index in Beijing strengthened, transforming Beijing into a center city. Until 2016, the urban hierarchy shows a pyramidal pattern in which Beijing, Shanghai, and Chengdu are the central cities, and Guangzhou, Wuhan, and Zhengzhou are the sub-central cities.

**Fig 4:**
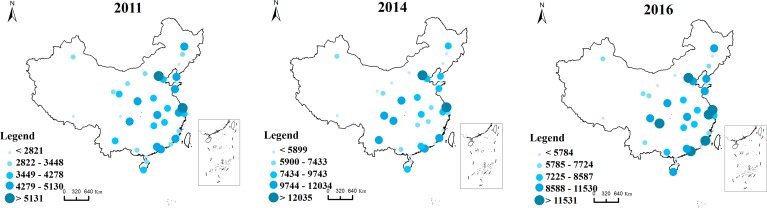
The receiving Baidu index among Chinese cities along “the Belt and Road”.

The map of the receiving Baidu index is presented in Fig 4. The results show that cities under this web search activity are on a spatial concentration trend that is mainly concentrated in the eastern coastal areas. Specifically, there are three main regions: the Beijing-Tianjin-Hebei, the Yangtze River Delta and the Pearl River Delta region. In contrast, in 2016, the receiving Baidu index in three regions accounts for 40% of the total, which indicates Beijing as the center of the Beijing-Tianjin-Hebei region, Shanghai as the center of the Yangtze River Delta region, and Guangzhou as the center of the Pearl River Delta region. This finding shows that the Beijing-Tianjin-Hebei, the Yangtze River Delta and the Pearl River Delta region are not only the focus of national socio-economic development but are also the regions gaining web search activity. At the same time, although cities such as Wuhan have a high level of economic development, surrounding cities have a low level of economic development. Therefore, we reasonably conclude that a significant spatial agglomeration in such a region has not yet formed.

In contrast, the sending and receiving Baidu indexes for the selected three years are largely correlated. That is, a city with a larger sending Baidu index tends to have a larger receiving Baidu index. Thus, a city that searches more for other cities is reciprocally searched for by other cities.

Some findings arise if the context of “the Belt and Road” is considered. Fig 1 shows several significant corridors distributed in central and east China, including Beijing-Shanghai, Beijing-Guangzhou, Beijing–Shenzhen, Beijing-Chengdu, and Beijing-Xi’an, with information flows in 2016. Connections among central and eastern cities have largely decreased, whereas those among western cities largely have largely increased since 2014. This result has two potential causes: (1) Internet users residing in western cities may have increased over the past several years. However, the number of internet users residing in western cities would negate this reason. According to the “Statistical Communique on National Economic and Social Development 2014” and the “Statistical Communique on National Economic and Social Development 2016”, the number of internet users from western cities (Xining, Lanzhou, Urumqi, Yinchuan and Kunming) was 30.56 million in 2014 and 29.96 million in 2016. (2) The search volume has increased in the previous several years, which indicates these cities have gained more connections with and attention from other cities. As a result, these significant corridors have weakened, which indicates that more cities, particularly western cities, have shared more opportunities.

### Ranking cities

#### Connection degree

**Fig 5:**
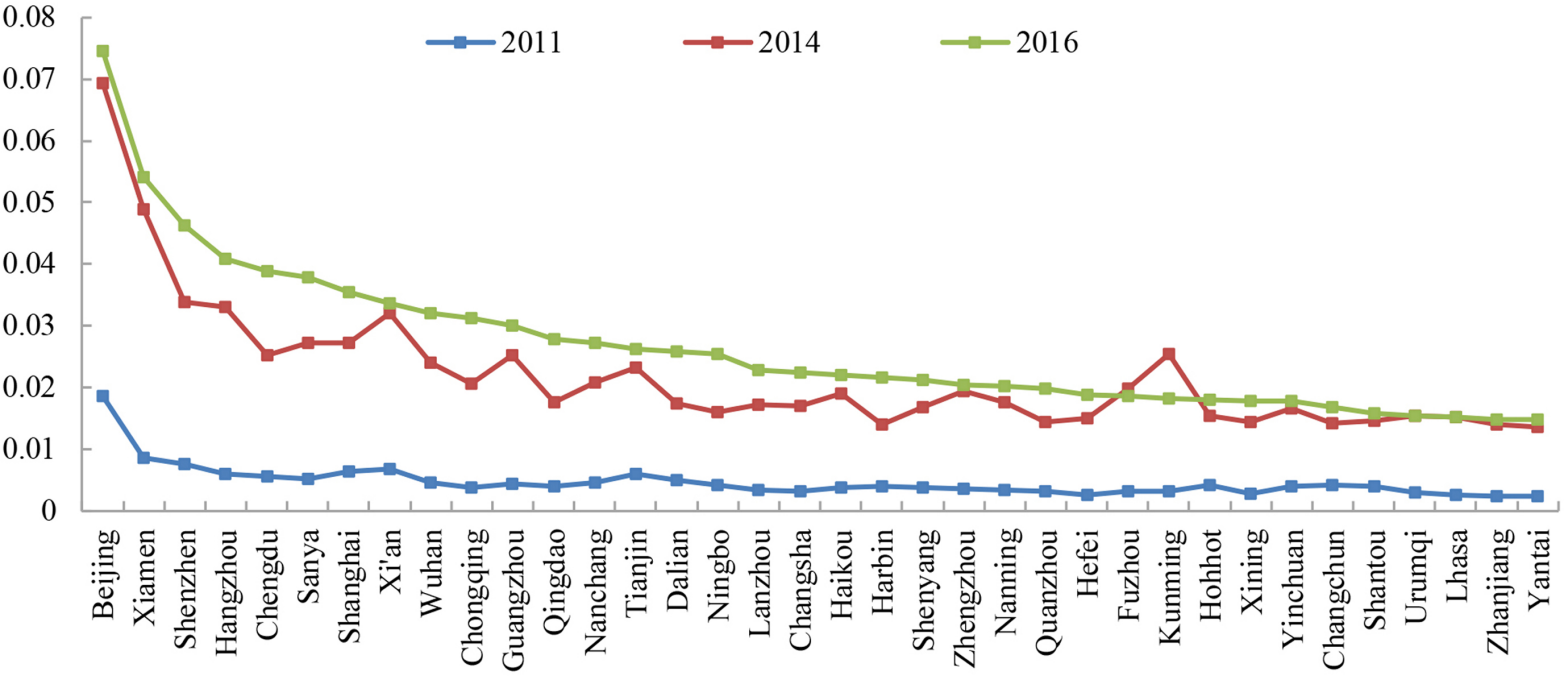
The rank of the connection degree of cities in the network (2011–2016).

“The Belt and Road” cities in China have significant centrality. In essence, the connection degree shows a substantial difference among cities (Fig 5).

As China’s political, economic and cultural center, Beijing has the strongest network connectivity with 0.074, which is higher than that of the secondary city of Xiamen. In addition, cities such as Shenzhen, Hangzhou, Chengdu, Sanya, Shanghai, Xi’an, Wuhan and Chongqing have similar and strong network connectivities, with connection degrees between 0.03 and 0.05. Cities such as Guangzhou, Qingdao, Nanchang, Tianjin, Dalian, Ningbo, Lanzhou, Changsha, Haikou, Harbin, Shenyang, Zhengzhou and Nanning have connection degrees between 0.02 and 0.03. Furthermore, cities such as Quanzhou, Hefei, Fuzhou and Kunming have lower connectivity degrees between 0.01 and 0.02. In addition, cities such as Zhanjiang and Yantai have the lowest connection degrees in the network.

The connection degree of cities has substantially improved from 2011, 2014 to 2016. For example, the connection degree of Beijing was only 0.0019 in 2011, however, it increased to 0.07 in 2014, which is more than three times the value in 2011. The connection degree of cities in 2014 showed some changes, in which the connection degree represents an abnormal value compared with the values in 2011 and 2016, the apex of which was Kunming with an increment of more than 8 times. A further examination of the Baidu index showed that Kunming significantly increased because of the violent incidents that occurred there in early March 2014, which explained the sudden increase in web search activity. However, the connection degree returned to a relatively normal state in 2016.

#### The urban hierarchy under web search activity

**Fig 6:**
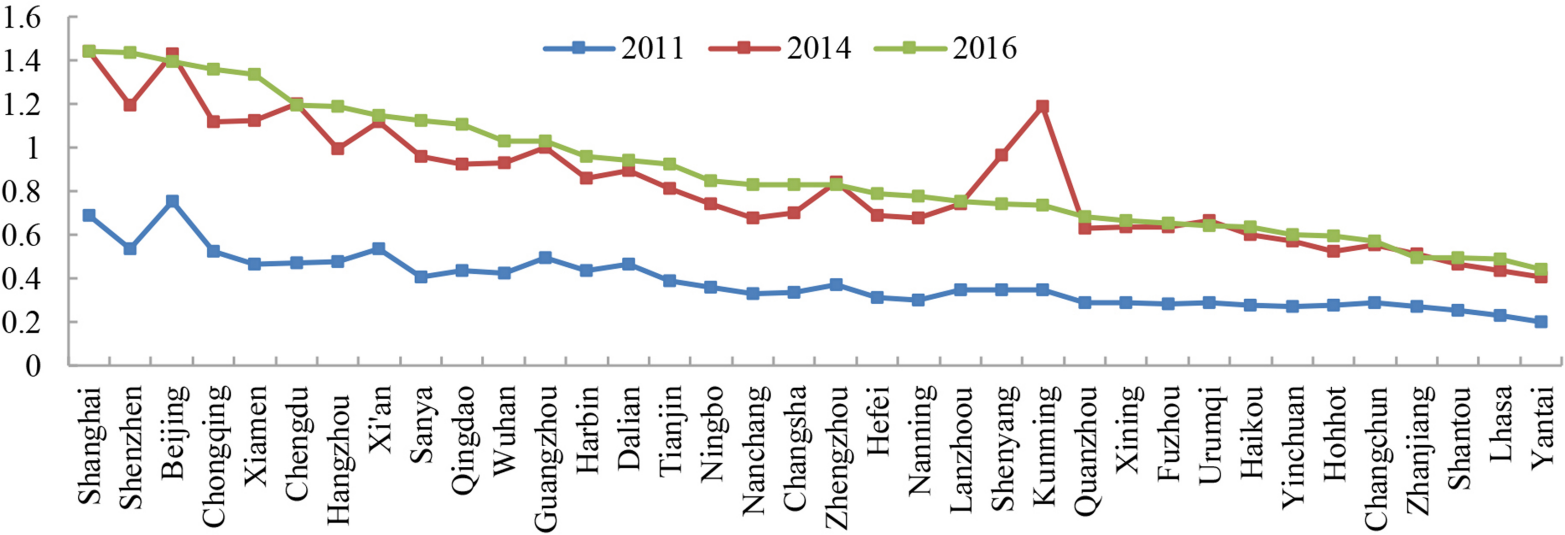
The rank of the external connection degree of cities.

As shown in the Fig 6, cities are ranked according to their external connection degree. Cities such as Shanghai, Shenzhen and Beijing have high external connection degrees. Cities such as Chongqing, Xiamen, Chengdu, Hangzhou, Xi’an, Sanya, Qingdao, Wuhan and Guangzhou have values that range from 1 to 1.5. Cities such as Harbin, Dalian, Hohhot and Changchun have external connection degrees from 0.5 to 1. Moreover, cities such as Shantou, Zhanjiang, Lhasa and Yantai have relatively low external connection degrees because of their geographical and administrative location.

Although discussions of urban hierarchy, in which “senior” cities have power over and control those below, may be unfashionable or unhelpful[57], it makes sense to examine cities in terms of urban rank[22]. The external connection degree is characterized by the city grade in urban networks. Thus, a higher external connection degree of a city is associated with a higher rank in the urban hierarchy of a city network. The urban hierarchy based on web search activity may be reflected using this indicator. Using the method of natural breaks, the 36 cities are divided into five classes (Table 3).

**Table 3:** The urban hierarchy and its evolution under web search activity.

Class	List of city in 2011	List of city in 2014	List of city in 2016
**National centers**	Beijing, Shanghai, Shenzhen	Shanghai, Beijing, Chengdu, Shenzhen, Xiamen	Shanghai, Shenzhen, Beijing, Chongqing, Xiamen, Chengdu
**National sub-centers**	Chengdu, Xi'an, Chongqing, Guangzhou, Hangzhou, Xiamen, Dalian, Harbin, Qingdao, Wuhan, Sanya	Chongqing, Xi'an, Hangzhou, Sanya, Wuhan, Qingdao, Dalian, Harbin, Kunming	Hangzhou, Xi'an, Sanya, Qingdao, Wuhan, Guangzhou, Harbin
**Regional centers**	Tianjin, Zhengzhou, Ningbo, Kunming, Lanzhou, Changsha, Nanchang, Hefei	Zhengzhou, Tianjin, Lanzhou, Ningbo, Changsha	Dalian, Tianjin, Ningbo, Nanchang, Changsha, Zhengzhou, Hefei, Nanning, Lanzhou
**Regional sub-centers**	Nanning, Changchun, Urumqi, Quanzhou, Xining, Fuzhou, Haikou, Hohhot, Yinchuan, Shantou,	Hefei, Nanning, Nanchang, Urumqi, Fuzhou, Xining, Quanzhou, Haikou, Yinchuan, Changchun, Hohhot, Shantou	Shenyang, Kunming, Quanzhou, Xining, Fuzhou, Urumqi, Haikou, Yinchuan, Hohhot, Changchun
**Local centers**	Lhasa,Shenyang,Yantai,Zhanjiang	Lhasa, Shenyang, Yantai, Zhanjiang	Zhanjiang, Shantou, Lhasa, Yantai

A shared opportunity for the 36 cities has been shown from the increase of national and regional centers since the implementation of “the Belt and Road” initiative. First, Beijing, Shanghai and Shenzhen are always in the list of national centers in 2016, and cities such as Chongqing, Xiamen, and Chengdu rank significantly as national centers. Second, the number of regional centers has increased since 2014. More regional sub-centers such as Hefei, Nanning and Nanchang have transformed into regional centers in 2016. Moreover, cities such as Guangzhou, Chongqing, and Qingdao are assumed to be regional centers. This phenomenon also verifies that the urban hierarchy based on web search activity is not exactly the same as the urban hierarchy based on physical and infrastructure links[58, 59]. Urban hierarchy based on web search activity is not entirely determined by the level of economic development.

**Fig 7:**
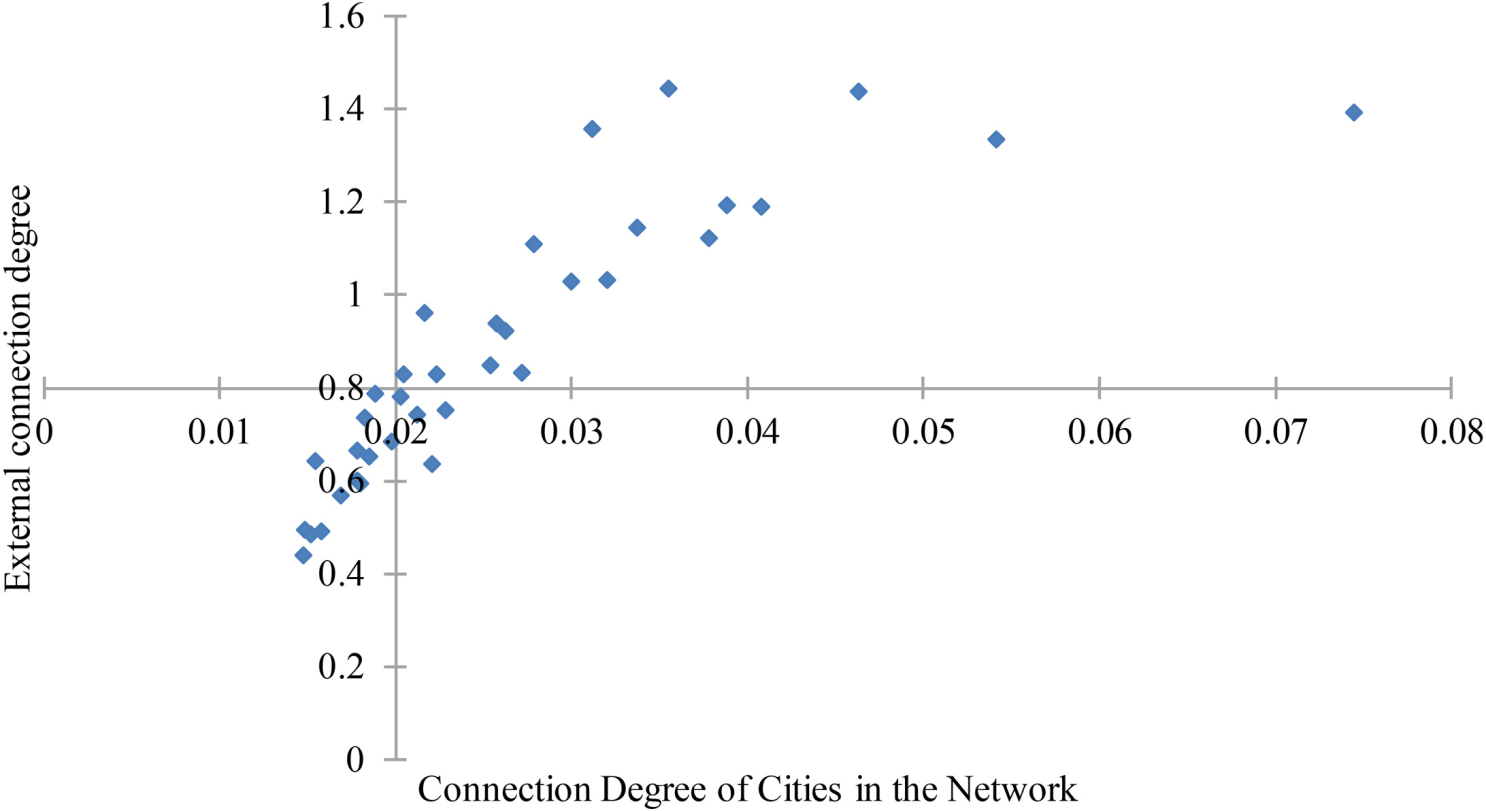
Quadrantal diagram of connection degree and external connection degree.

The connection degree is consistent with the external connection degree of cities. As shown in Fig 7, the abscissa presents the connection degree, and the ordinate shows the external connection degree. The connection degree has a positive correlation with the external connection degree of cities. Thus, the performance of a city with a high hierarchy in the network system tends to have a high connection degree. The connection degree increases when the urban hierarchy of a city increases. However, there are some exceptions, as the connection degree is not always consistent with the external connection degree, such as Shanghai, Chongqing, Nanchang, Harbin, and Urumqi. Cities such as Shanghai, Chongqing, Harbin, Hefei, Kunming and Urumqi have a higher urban hierarchy than their connection degrees indicate. In contrast, cities such as Sanya, Nanchang, Ningbo and Haikou have higher connection degrees than their urban hierarchy because that these cities always have strong connectivity in a small area, even if they have a low urban hierarchy in the network system.

### The verification of influencing factors under web search activity

#### The effect of economy

**Fig 8:**
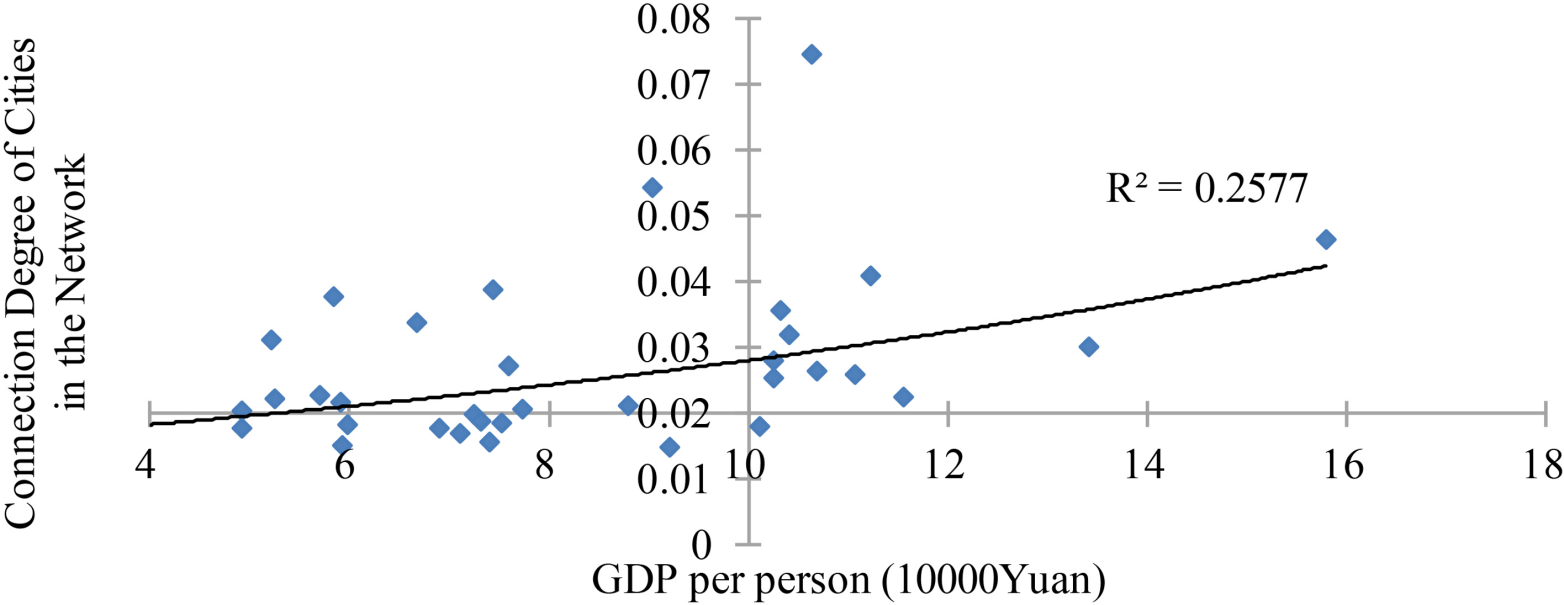
Quadrantal diagram of GDP per person and connection degree.

The connection degree under web search activity is not influenced by its own economic development. As shown in Fig 8, the abscissa presents the GDP per person, and the ordinate presents the connection degree. To a certain extent, the connection degree and GDP per person have a correlation. However, this correlation does not reach statistical significance, with a regression coefficient of 0.2577 in 2016.

These results show that there is no obvious correlation between urban network connectivity and economic development. However, what about the correlation between the connection degree and economic gap? Will the web search activity tend to occur between cities with similar economic development or cities with a large economic gap?

**Fig 9:**
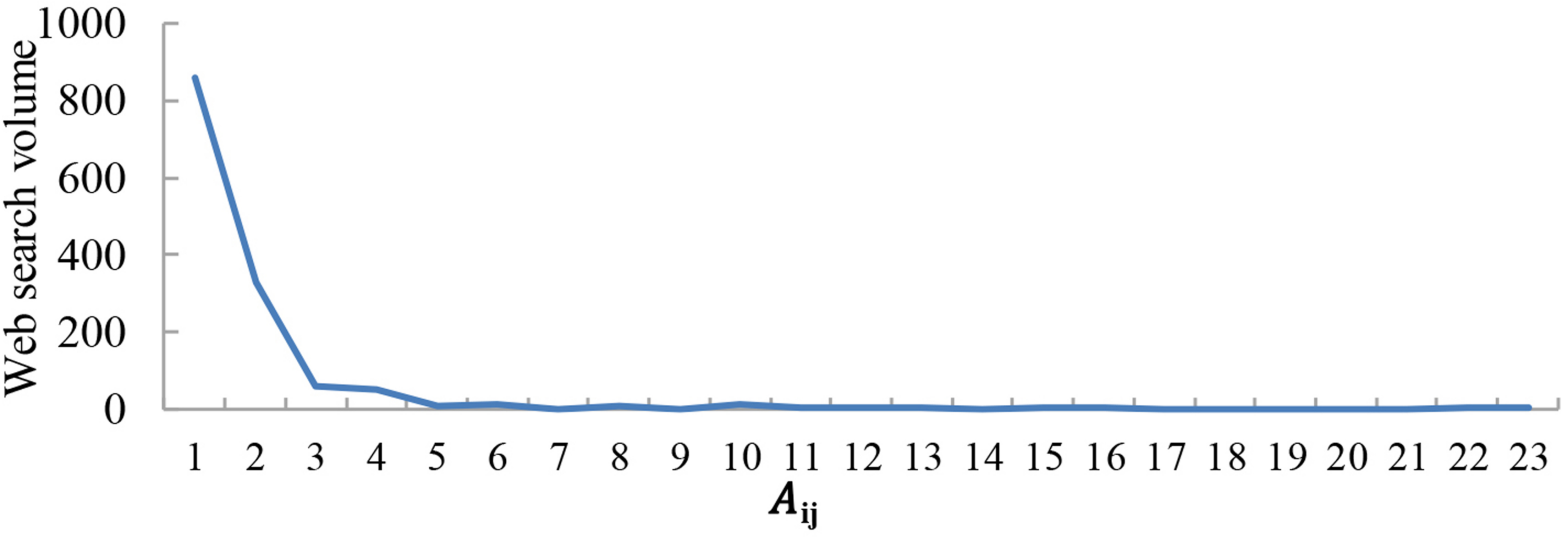
Web search activity under a different economic gap in 2011.

**Fig 10:**
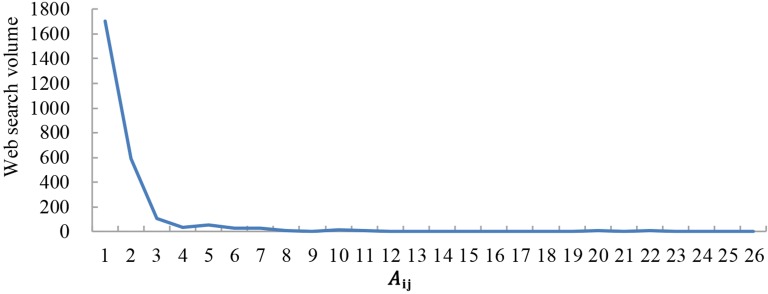
Web search activity under a different economic gap in 2014.

**Fig 11:**
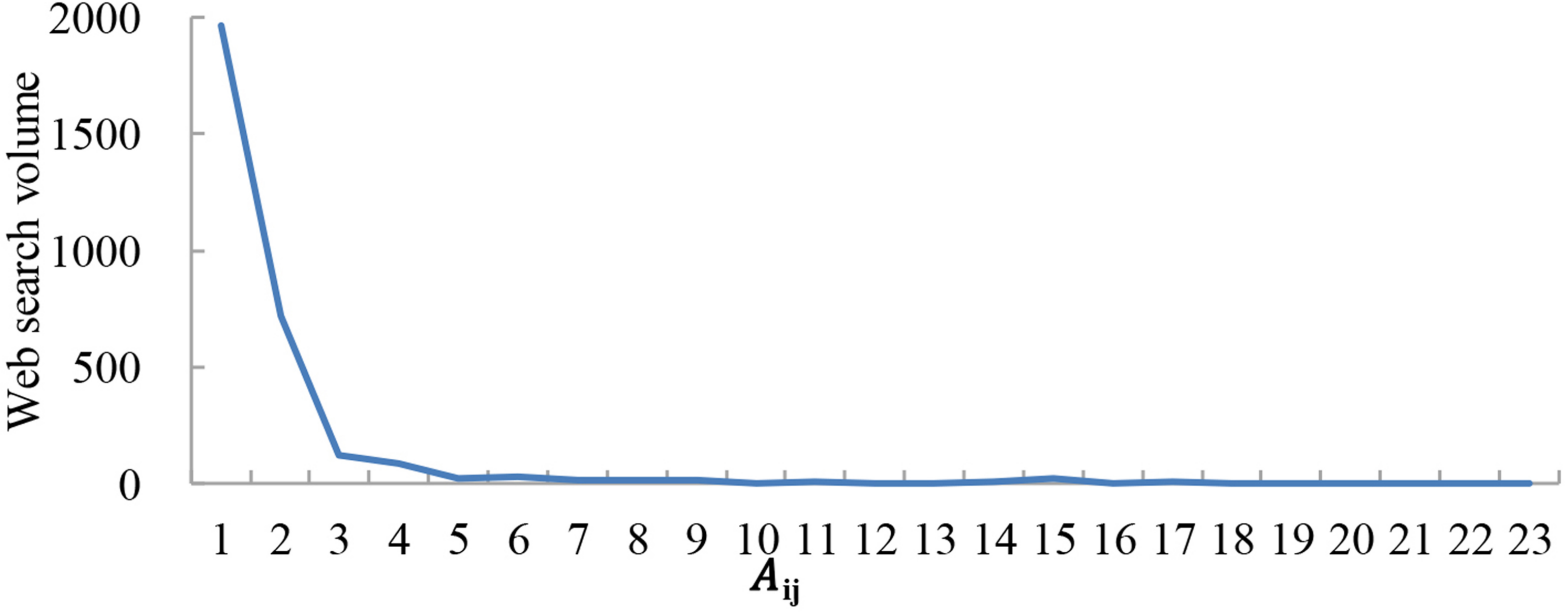
Web search activity under a different economic gap in 2016.

The results indicate that web search activity tends to occur between cities with a large economic gap. As shown in Figs 9–11, the abscissa is A_ij_, which presents the economic gap between cities, and the ordinate is the web search activity. The results show that web search activity tended to occur between cities with a large economic gap in 2011, 2014, and 2016. Thus, when the A_ij_ value is lower, both the economic gap and web search activity are also lower. With an increase in A_ij_, the economic gap between cities decreases, which causes a reduction in web search activity.

The web search activity under the same economic gap has been increasing yearly. When A_ij_≈0, the economic gap between cities is large; the value of the web search activity was 860 in 2011, 1700 in 2014 and 1965 in 2016.

#### The effect of geographical proximity

**Fig 12:**
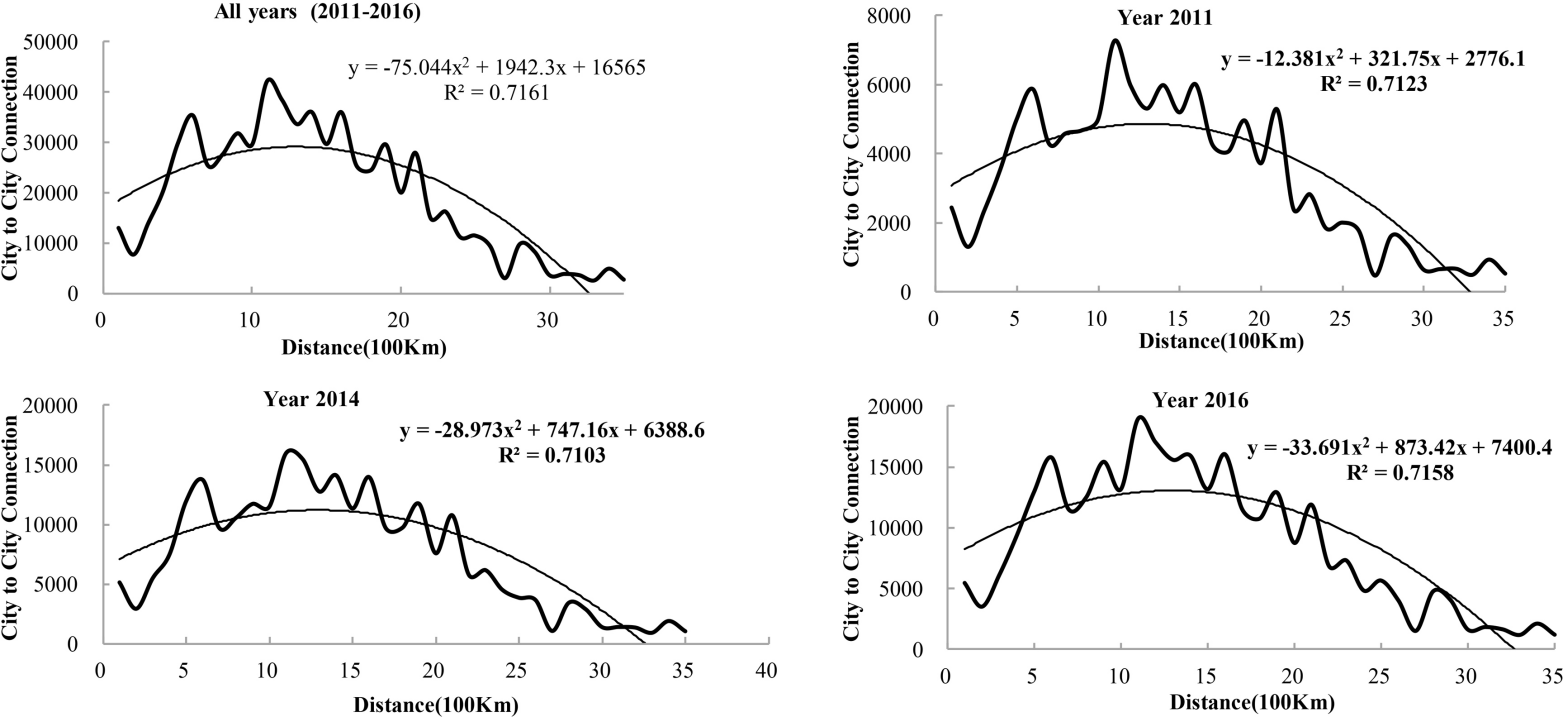
Effect and historic evolution of spatial distance on web search activity from 2011 to 2016.

To explore the relationship between geographical proximity and web search activity and the change that occurs in this relationship, a fitting analysis of inter-city web search activity at different distances is undertaken (Fig 12). Each fitting function is constructed using data on the amount of inter-city web search activity in 35 distance intervals across the 3 years that comprised the study period (2011, 2014 and 2016). By examining the amount of inter-city web search activity at each distance interval, a fitting function is obtained: Y = -75.044x^2^ + 1942.3x + 16,565. This is a quadratic equation with a negative slope and a relatively high fitting degree of 0.7161 (R^2^ = 0. 7161). The results show that within a certain distance range, the increment of distance leads to an increment of web search activity between cities. However, this increasing process is quite limited and begins to decrease beyond a certain range of distance. The result indicates that the distribution of city nodes is related to distance. Specifically, nodes closer to the city are associated with greater inter-city web search activity, whereas nodes farther away are associated with less web search activity. From the fitting function and its correlation coefficient (R^2^) for each year, the impact of geographical proximity on inter-city web search activity has the same trend. For the years of 2011, 2014 and 2016, the correlation coefficients (R^2^) are 0.7123, 0.7103 and 0.7158, respectively, which also indicates a strong correlation between distance and web search activity.

In this paper, the largest web search activity among cities occurs within the distance of 1000–1500 km, accumulated to 34,937 among selected years, whereas the least amount of inter-city web search activity occurs within a distance of 3,300–3,500 km, accumulated to 3,443 among selected years.

#### The effect of administrative rank

As previously discussed, the large search volumes between cities occur in the range of 1000–1500 km; 187 total links among cities occur in this distance. Among these links, 178 links based on at least one provincial capital city occurred, accounting for 95%. Moreover, 115 links between provincial capitals occur, accounting for 62%. Based on these data, it is tentatively speculated that web search activity is significantly influenced by administrative rank. This impact is estimated as follows.

Among the 36 cities selected, 26 cities are provincial capitals, and 10 cities are non-capital cities. Therefore, 650 links between provincial capital cities and 260 links based on at least one provincial capital city are supposed to occur. Accordingly, 36 links based on at least one provincial capital city and 4 links between non-capital cities are expected. However, the results show that large search volumes tend to occur among provincial capital cities (Table 4). In the first 250 links, no fewer than 47 links are based on provincial capital cities in each of the 50 links. For example, in the first 50 of the search volume, no fewer than 47 pairs of provincial-based links occur in each year; only 2 or 3 links occur between non-provincial nodes. Thereafter, for every 50 pairs in the search volume, there are no fewer than 43 pairs of links based on at least one provincial capital city, which is substantially higher than the expected 36 links. Therefore, 20 links based on non-capital cities are expected in the top 250. In reality, only 16 links are based on non-capital cities in 2014. This finding suggests that inter-city links based on web search activity are associated with a city’s administrative rank. This correlation indicates that a high administrative rank city tends to have more opportunities for higher web search activity among cities.

**Table 4:** The expected and actual web search activities in top 250.

The rank of web search activity	Based on at least one provincial capital city				Non-capital to non-capital cities			
	Expected links	Actual links			Expected links	Actual links		
		2011	2014	2016		2011	2014	2016
**1–50**	36	48	48	47	4	2	2	3
**51–100**	36	46	45	45	4	4	5	5
**101–150**	36	46	44	43	4	4	6	7
**151–200**	36	46	47	47	4	4	3	3
**201–250**	36	47	50	49	4	3	0	1
**Total**	180	233	234	231	20	17	16	19

## Discussion

“The Belt and Road” is expected to facilitate interactions among numerous city centers. This exploratory study would contribute to the literature on “the Belt and Road” by providing a systematic assessment of the urban hierarchies for Chinese cities along “the Belt and Road”. Furthermore, with the rapid development of internet technology and increase in Chinese internet user scale, the internet has become an important window to show inter-city links in China. The study introduces the Baidu database as a comparative analysis of information flows in China, which is an effective supplement to the existing research. Specifically, the Baidu index represents an under-utilized database of information that has been characterized as geo-tagged and massive. The proposed research is specifically concerned with how information flows are merged and the specific opportunities that are offered for Chinese cities along “the Belt and Road”. Therefore, research on cities in this area would not only be helpful to guide the long-term development of “the Belt and Road” but would also make economic centers more attractive by providing productivity benefits.

Overall, the results show that urban networks have undergone continuous adjustment, in which web search activity experienced a process of weakening between certain cities but strengthening between other cities. Some cities, especially western cities in China, have shared more opportunity since the initiative of “the Belt and Road”. Our research also confirm that there are significant urban hierarchies under informational flows among cities along “the Belt and Road”. Our research also examine some factors that could influence the urban network based on web search activity. (1)The results show that it is influenced more by the economic gap between cities than by the economic development of its own city. In other words, cities with a large economic gap tend to be more attractive for high web search activity. This finding can be explained by the traditional theory of flow space that cities with large economic gaps tend to have large potential energy differences. As a result, more elements flow among cities, and cities become more attractive. (2)The web search activity is affected by geographical proximity, which is consistent with the "distance decay". Nodes closer to the city are associated with greater inter-city web search activity, whereas nodes farther away are associated with less web search activity. Thus, the development of information technology does not cause the demise of geospatial relationships. Why is web search activity affected by geographical proximity? Traditional geographical proximity plays an important role in searching for other cities by network users. The reason is that although information dissemination on the internet is no longer affected by the friction of distance cost, the searching behavior for information is remain limited by real life. These limitations cause a relationship increase to occur in neighboring cities. (3)The web search activity is a selection process for administrative rank. Regression models indicate that conditioning on administrative characteristics is positively associated positively with a high degree of informational flows in China. The distance among provincial cities is always larger than 600 km. For this reason, web search activity increases consistent with a distance increase in the range of 0 to 600 km. This finding is consistent with the previous research results that urban networks of information flows/exchange present as an altogether messier and more dynamic amalgam of hierarchical characteristic [22].

This paper discusses the web search activity, connection degree, urban hierarchy and its influencing factors, which is a new attempt to investigate not only “the Belt and Road” but also urban networks under the influence of information flows. The results also demonstrate the effectiveness of using web search activity to study urban network systems. Our analysis suffers from several limitations, which indicate potential directions for future research. First, web search activity is vulnerable to emergencies; for example, we cannot ignore the fact that in some years, cities that are affected by emergencies, such as the violent incidents in Kunming had a higher web search activity and a greater connection degree in network. Second, web search activity is influenced not only by the economy, distance, and administrative rank of a city but also factors such as infrastructure construction and industrial division. Third, a proportion of the web search volume without a semantic connection cannot be excluded. Therefore, it is necessary to introduce multivariate assessments.

## Supporting information

S1 Table Baidu index in 2011. (PDF)

S2 Table Baidu index in 2014. (PDF)

S3 Table Baidu index in 2016. (PDF)

## References

[1] Tao M. Feature: China-built power grid shines hearts of grass roots in Egypt 2016 [updated July 19,2016; cited 2016]. Available from: http://news.xinhuanet.com/english/2016-07/19/c_135525281.htm.

[2] Tian S. Spotlight: Putin eyes closer partnership with China along Silk Road and beyond 2016 [updated June 23,2016; cited 2016]. Available from: http://news.xinhuanet.com/english/2016-06/23/c_135461178.htm.

[3] Kunaka C, Carruthers R. Trade and Transport Corridor Management Toolkit. Washington, DC: World Bank; 2014.

[4] Zhao Y, Guo J, Zhao X, Tang L, Wang Y. The characteristics of city network along "Belt and Road" in China based on railway and aviation passenger transport. Journal of Arid Land Resources and Environment. 2017;31(01):51–7.

[5] Allen J. Cities of power and influence: settled formations: Routledge, New York; 1999.

[6] Allen J. Powerful city networks: more than connections, less than domination and control. Urban studies. 2010;47(13):2895–911.

[7] Castells M. The rise of the network society Oxford: Blackwell; 1996.

[8] Graham S. Global grids of glass: on global cities, telecommunications and planetary urban networks. Urban Studies. 1999;36(5–6):929–49.

[9] Ota M, Fujita M. Communication technologies and spatial organization of multi-unit firms in metropolitan areas. Regional science and urban economics. 1993;23(6):695–729.

[10] Taylor PJ, Catalano G, Walker DRF. Measurement of the World City Network. Urban Studies. 2002;39(13):2367–76.

[11] Choi JH, Barnett GA, Chon BS. Comparing world city networks: a network analysis of Internet backbone and air transport intercity linkages. Global Networks A Journal of Transnational Affairs. 2006;6(1):81–99.

[12] Derudder B, Witlox F. An Appraisal of the Use of Airline Data in Assessing the World City Network: A Research Note on Data. Urban Studies. 2005;42(13):2371–88.

[13] Dicken P, Kelly PF, Olds K, Yeung WC. Chains and networks, territories and scales: towards a relational framework for analysing the global economy. Global Networks. 2001;1(2):89–112.

[14] Timberlake M. The polycentric metropolis: learning from mega-city regions in Europe. Journal of the American Planning Association. 2008;16(2006):384–5.

[15] Vijver EVD, Derudder B, Witlox F. Air passenger transport and regional development: Cause and effect in Europe. Promet—Traffic—Traffico. 2016;28(2).

[16] Järv O, Müürisepp K, Ahas R, Derudder B, Witlox F. Ethnic differences in activity spaces as a characteristic of segregation: A study based on mobile phone usage in Tallinn, Estonia. Urban Studies. 2015;52:págs. 2680–98.

[17] Liu X, Derudder B, Wu K. Measuring polycentric urban development in China: An intercity transportation network perspective. Regional Studies. 2016;50(8):1302–15.

[18] Derudder B, Taylor PJ, Hoyler M, Ni P, Liu X, Zhao M, et al Measurement and interpretation of connectivity of Chinese cities in world city network, 2010. Chinese Geographical Science. 2013;23(3):261–73.

[19] Summers T. China’s ‘New Silk Roads’: sub-national regions and networks of global political economy. Third World Quarterly. 2016;37(9):1628–43.

[20] Taylor P. World cities in globalization. GaWC Research Bulletin. 2008;263:1–19.

[21] Beaverstock JV, Doel MA, Hubbard PJ, Taylor PJ. Attending to the world: competition, cooperation and connectivity in the World City network. Global networks. 2002;2(2):111–32.

[22] Boulton A, Devriendt L, Brunn SD, Derudder B, Witlox F. City networks in cyberspace and time: using Google hyperlinks to measure global economic and environmental crises ICTs for Mobile and Ubiquitous Urban Infrastructures: Surveillance, Locative Media and Global Networks: IGI Global; 2011 p. 67–87.

[23] Grubesic TH, Matisziw TC. A spatial analysis of air transport access and the essential air service program in the United States. Journal of Transport Geography. 2011;19(1):93–105.

[24] Castells M. The Rise of Informational Society (2nd edn). Oxford: Basil Blackwell; 2001.

[25] Grace J, Kenny C, Qiang CZ-W. Information and communication technologies and broad-based development: partial review of the evidence Washington, DC: World Bank Publications; 2004.

[26] Batty M. Virtual geography. Futures. 1997;29(4):337–52.

[27] Kellerman A. Fusions of information types, media and operators, and continued American leadership in telecommunications. Telecommunications Policy. 1997;21(6):553–64.

[28] Devriendt L, Derudder B, Witlox F. Cyberplace and cyberspace: two approaches to analyzing digital intercity linkages. Journal of Urban Technology. 2008;15(2):5–32.

[29] Zook M. The geographies of the Internet. Annual review of information science and technology. 2006;40(1):53–78.

[30] Adams PC. Cyberspace and virtual places. Geographical Review. 1997;87(2):155–71.

[31] Zook MA. Old Hierarchies or New Networks of Centrality? The Global Geography of the Internet Content Market. American Behavioral Scientist. 1999;44(10):1679–96.

[32] Townsend AM. Network Cities and the Global Structure of the Internet. American Behaviouralentist. 2001;44(44):1697–716.

[33] Moss ML, Townsend A. Tracking the net: Using domain names to measure the growth of the internet in U.S. cities. Journal of Urban Technology. 1997;4(3):261–81.

[34] Malecki EJ. The economic geography of the Internet's infrastructure. Economic geography. 2002;78(4):399–424.

[35] Rutherford J, Gillespie A, Richardson R. The territoriality of pan-European telecommunications backbone networks. Journal of Urban Technology. 2004;11(3):1–34.

[36] Burch H, Cheswick B. Mapping the internet. Computer. 1999;32(4):97–8.

[37] Wheeler DC, O'Kelly ME. Network topology and city accessibility of the commercial Internet. The Professional Geographer. 1999;51(3):327–39.

[38] Choi JH, Barnett GA, CHON BS. Comparing world city networks: a network analysis of Internet backbone and air transport intercity linkages. Global Networks. 2006;6(1):81–99.

[39] Devriendt L, Derudder B, Witlox F. Conceptualizing digital and physical connectivity: The position of European cities in Internet backbone and air traffic flows. Telecommunications Policy. 2010;34(8):417–29.

[40] Hillis K. On the margins: the invisibility of communications in geography. Progress in Human Geography. 1998;22(4):543–66.

[41] Batty M. Editorial. SAGE Publications Sage UK: London, England; 1990.

[42] Grubesic TH, Murray AT. Geographies of imperfection in telecommunication analysis. Telecommunications Policy. 2005;29(29):69–94.

[43] Greenstein S. Data Constraints & the Internet Economy: Impressions & imprecision. Motion Imaging Journal Smpte. 2007;117(7):32–5.

[44] Short JR, Kim Y, Kuus M, Wells H. The dirty little secret of world cities research: data problems in comparative analysis. International Journal of Urban and Regional Research. 1996;20(4):697–717.

[45] Williams JF, Brunn SD. Cybercities of Asia: measuring globalization using hyperlinks (Asian cities and hyperlinks). Asian Geographer. 2004;23(1–2):121–47.

[46] Terlouw K, Denkers R. The geography of regional websites: Regional representation and regional structure. Geoforum. 2011;42(5):578–91.

[47] Long M, Sun G, Ma L, Wang J. An Analysis on the Variation Between the Degree of Consumer Attention of Travel Network and Tourist Flow in Regional Tourism:A Case of Sichuan Province. Areal Research & Development. 2011;30(3):93–7.

[48] CNNIC. Statistical Report on China 's Internet Development. http://www.cnnic.net.cn/.

[49] Liebelson D. Map: Here are the countries that block facebook, twitter, and youtube. Mother Jones. 2014.

[50] National Development and Reform Commission MoFA, and Ministry of, China CotPsRo. Vision and Actions on Jointly Building Silk Road Economic Belt and 21st-Century Maritime Silk Road 2015.

[51] Dai L, Derudder B, Liu X, Ye L, Duan X. Simulating infrastructure networks in the Yangtze River Delta (China) using generative urban network models. Belgeo Revue belge de géographie. 2016(2).

[52] Nannan Z, Chaolin G. From geographical space to composite space-The Urban Space under the Influence of Information Networks. Human Geography. 2002;17(4):20–4.

[53] Pred AR. City systems in advanced economies: Past growth, present processes, and future development options London: Hutchinson London; 1977.

[54] Junde L. Perspective of the "administrative region economy" phenomenon in China's transitional period-an introduction of human-economic geography with Chinese characteristic. Economic Geography. 2006;6:000.

[55] Pan F, Xia Y, Liu Z. The relocation of headquarters of public listed firms in China: A regional perspective study. Acta Geographica Sinica. 2013;68(4):449–63.

[56] Wall R. Netscape: cities and global corporate networks2009.

[57] Taylor PJ. Urban economics in thrall to Christaller: a misguided search for city hierarchies in external urban relations. Environment and Planning A. 2009;41(11):2550–5.

[58] Zhong Y, Lu Y. Hierarchical structure and distribution pattern of Chinese urban system based on railway network. Geograph Res. 2011;30:785–94.

[59] Lu L, Huang R. Urban hierarchy of innovation capability and inter-city linkages of knowledge in post-reform China. Chinese Geographical Science. 2012;22(5):602–16.

