# Correction: Urban networks among Chinese cities along "the Belt and Road": A case of web search activity in cyberspace

**DOI:** 10.1371/journal.pone.0196141

**Published:** 2018-04-17

**Authors:** Lu Zhang, Hongru Du, Yannan Zhao, Rongwei Wu, Xiaolei Zhang

There are errors in the Author Contributions. The correct contributions are: Conceptualization: LZ. Data curation: LZ. Formal analysis: LZ. Funding acquisition: HD. Methodology: LZ RW. Project administration: HD. Resources: LZ. Software: LZ YZ. Visualization: LZ YZ. Writing–original draft: LZ. Writing–review & editing: LZ HD YZ XZ.
