# Supplementary material for: Urban networks among Chinese cities along "the Belt and Road": A case of web search activity in cyberspace
Source: PLoS One. 2017 Dec 4;12(12):e0188868. doi: 10.1371/journal.pone.0188868 (PMC5714330; doi:10.1371/journal.pone.0188868)
Supplement: S1 Table — (PDF) [file pone.0188868.s001.pdf]

S1 Table. Baidu index in 2011

	CQ	SH	FZ	GZ	HZ	HK	NN	KM	LS	HB	CC	SY	HH	XN	YC	LZ	XA	UM	CD	ZZ	WH	CS	NC	HF	TJ	NB	SZ	ZJ	ST	QD	YT	DL	XM	QZ	SY	BJ
**CQ**	1887	345	112	218	160	94	101	235	65	101	97	103	80	63	70	88	217	95	312	177	183	130	104	114	153	127	254	61	69	99	75	99	112	115	39	528
**SH**	182	3808	172	276	361	110	132	143	59	171	151	172	121	68	85	106	228	122	179	312	245	158	171	212	275	262	303	67	92	165	111	165	150	152	48	786
**FZ**	73	161	887	121	92	60	75	63	13	64	63	67	52	24	40	57	76	52	74	85	99	73	75	71	80	76	104	28	56	67	56	66	135	131	11	196
**GZ**	126	254	111	1590	146	118	134	100	42	107	95	104	78	57	65	75	136	83	126	163	181	152	117	100	139	107	380	92	118	96	73	105	106	103	49	377
**HZ**	111	544	130	191	1796	76	111	88	31	111	96	106	78	51	62	73	140	77	117	176	162	115	123	140	160	248	180	54	69	110	77	108	115	102	31	517
**HK**	73	116	66	113	77	393	66	66	13	67	63	64	56	30	35	53	74	58	73	78	77	71	66	63	75	66	94	54	50	61	46	61	63	62	60	160
**NN**	82	166	76	153	100	66	755	77	14	73	70	73	57	33	44	57	91	62	88	95	101	85	75	73	93	77	137	58	58	71	58	71	74	71	15	227
**KM**	131	188	78	144	108	70	87	904	42	79	72	80	63	47	55	66	113	67	134	117	110	87	78	80	107	82	132	50	61	77	63	77	77	73	24	282
**LS**	75	140	59	92	76	50	54	54	750	57	55	58	57	36	51	66	105	85	79	100	77	64	56	64	78	59	90	19	37	59	49	55	58	54	5	236
**HB**	101	265	86	163	133	74	75	80	29	1874	208	182	112	47	60	67	122	68	104	135	118	91	81	95	246	98	151	46	62	108	77	154	82	78	35	569
**CC**	71	152	87	106	63	59	59	61	14	133	982	118	79	31	41	52	79	58	73	86	81	70	64	69	152	71	99	28	49	71	61	98	66	63	10	280
**SY**	98	251	85	157	130	73	75	79	35	980	145	1087	91	50	62	68	134	73	99	1469	125	90	82	96	185	97	160	48	62	96	73	119	82	79	29	461
**HH**	71	136	64	92	82	57	55	61	22	76	75	82	1191	30	62	61	94	59	77	87	80	68	62	67	138	66	89	21	42	70	57	72	61	59	9	357
**XN**	80	125	64	94	80	57	59	62	74	63	64	64	59	254	59	85	130	62	86	92	83	69	60	65	82	65	90	18	37	66	55	63	62	57	7	205
**YC**	75	126	62	92	80	54	56	62	21	67	62	66	83	49	469	82	136	64	78	103	79	67	61	63	87	64	90	21	39	65	51	64	61	59	8	233
**LZ**	92	170	74	114	97	65	65	69	56	73	69	75	70	77	82	639	215	83	97	107	95	79	72	74	100	75	111	36	56	74	63	71	70	71	18	253
**XA**	164	366	111	205	168	84	92	101	58	119	104	122	112	79	106	143	1804	117	164	269	176	120	106	122	192	121	323	56	68	119	85	115	100	93	29	621
**UM**	85	150	69	102	86	60	64	64	24	67	65	68	67	46	61	76	115	999	89	110	87	74	66	74	88	69	100	29	47	69	59	65	68	64	15	246
**CD**	223	385	107	177	186	196	94	85	33	112	91	99	75	50	60	69	131	73	974	153	138	104	100	110	144	169	158	55	66	95	71	97	100	89	18	438
**ZZ**	94	236	84	151	127	71	74	78	40	86	81	88	69	53	63	68	145	77	94	2803	131	88	83	96	124	96	168	49	62	84	69	84	81	80	22	352
**WH**	117	319	100	218	153	76	85	85	37	89	83	91	71	52	61	71	132	73	122	185	1136	138	112	125	139	112	236	53	66	91	71	93	93	90	25	418
**CS**	87	177	82	185	101	69	77	76	25	77	71	75	62	43	51	62	93	65	84	105	153	969	103	81	101	79	197	48	57	70	61	78	76	72	24	277
**NC**	86	207	102	149	123	69	73	73	24	74	72	75	64	44	50	60	97	62	92	107	121	92	906	90	101	90	155	45	62	73	61	72	83	81	19	266

**HF**	81	290	77	118	131	61	64	66	25	72	69	72	60	38	45	59	92	60	80	104	150	78	80	1334	109	95	128	30	48	80	60	83	73	69	11	293
**TJ**	92	224	82	130	111	72	71	73	28	116	101	114	92	48	63	70	126	73	91	149	113	84	78	89	1877	85	137	43	59	104	76	105	80	73	21	786
**NB**	95	509	87	139	281	66	72	69	18	81	76	80	63	38	58	64	99	64	90	119	125	85	91	92	106	1062	135	43	62	86	66	85	88	79	18	296
**SZ**	150	327	149	441	166	108	128	107	43	115	105	112	80	56	67	79	164	87	149	186	233	160	146	118	153	129	2334	77	121	103	76	114	123	116	45	528
**ZJ**	65	130	49	82	66	40	44	44	56	47	45	48	47	26	41	56	95	75	69	90	67	54	46	54	68	49	80	450	27	49	39	45	48	44	30	226
**ST**	77	134	69	225	80	59	64	62	8	58	57	62	43	20	27	39	80	44	76	88	79	79	66	60	77	66	167	56	588	64	47	62	81	71	7	181
**QD**	106	325	90	159	147	70	77	80	30	120	103	117	84	58	68	76	141	77	112	186	149	95	87	109	214	109	149	50	63	1335	162	111	84	80	17	573
**YT**	55	120	39	72	56	30	34	34	46	37	35	38	37	16	31	46	85	65	59	80	57	44	36	44	58	39	70	60	17	39	907	35	38	34	20	216
**DL**	108	312	93	162	147	72	78	82	28	225	176	312	101	49	65	72	128	76	111	156	135	98	85	99	192	104	156	45	64	115	96	1212	88	82	22	615
**XM**	113	416	248	243	207	76	86	85	28	90	82	89	70	51	61	70	124	72	117	142	168	112	117	99	136	163	195	57	91	88	67	89	1069	279	27	411
**QZ**	71	147	168	115	92	63	65	64	15	65	63	66	57	25	41	56	76	54	72	87	94	71	71	67	83	75	109	33	64	68	57	68	157	897	8	189
**SY**	121	226	84	162	129	315	77	81	35	112	86	91	71	48	57	65	122	69	132	129	114	93	76	80	127	89	136	56	62	80	64	86	84	76	151	358
**BJ**	197	516	156	291	230	118	138	146	60	234	199	224	212	74	101	121	274	139	193	372	245	174	155	177	657	167	291	70	92	189	130	193	134	138	48	4280

[a] *CQ: Chongqing; SH: Shanghai; FZ: Fuzhou; GZ: Guangzhou; HZ: Hangzhou; HK: Haikou; NN: Nanning; KM: Kunming; LS: Lhasa; HB: Harbin; CC: Changchun; SY: Shenyang; HH: Hohhot; XN: Xining; YC: Yinchuan; LZ: Lanzhou; XA: Xi'an; UM: Urumqi; CD: Chengdu; ZZ: Zhengzhou; WH: Wuhan; CS: Changsha; NC: Nanchang; HF: Hefei; TJ: Tianjin; NB: Ningbo; SZ: Shenzhen; ZJ: Zhanjiang; ST: Shantou; QD: Qingdao; YT: Yantai; DL: Dalian; XM: Xiamen; QZ: Quanzhou; SY: Sanya; BJ: Beijing*

[b] *A few of default values have been interpolated according to the values in other years.*
