# Supplementary material for: Urban networks among Chinese cities along "the Belt and Road": A case of web search activity in cyberspace
Source: PLoS One. 2017 Dec 4;12(12):e0188868. doi: 10.1371/journal.pone.0188868 (PMC5714330; doi:10.1371/journal.pone.0188868)
Supplement: S2 Table — (PDF) [file pone.0188868.s002.pdf]

S2 Table. Baidu index in 2014

	CQ	SH	FZ	GZ	HZ	HK	NN	KM	LS	HB	CC	SY	HH	XN	YC	LZ	XA	UM	CD	ZZ	WH	CS	NC	HF	TJ	NB	SZ	ZJ	ST	QD	YT	DL	XM	QZ	SY	BJ
**CQ**	2975	633	287	640	503	181	229	326	134	220	188	204	176	140	143	218	505	204	848	387	485	321	238	301	390	219	508	124	145	214	138	181	244	229	75	903
**SH**	391	5462	404	638	1103	213	301	328	109	335	276	309	235	150	162	253	489	236	466	629	555	406	386	614	535	436	659	143	182	319	186	274	320	290	91	1401
**FZ**	161	321	1695	270	265	126	148	142	25	141	128	139	110	70	79	122	177	121	184	196	211	171	193	176	180	152	226	75	119	136	100	130	372	335	23	364
**GZ**	252	467	376	2573	383	207	306	223	66	205	176	190	156	114	124	171	275	167	304	329	378	356	264	262	296	184	804	179	213	185	132	170	237	206	87	670
**HZ**	243	1004	300	479	2997	158	202	202	61	231	194	218	168	112	123	172	306	159	282	389	363	278	282	398	387	386	373	115	140	228	143	204	234	204	68	937
**HK**	155	241	143	368	196	847	150	145	27	143	129	133	118	72	74	117	163	119	169	178	187	157	139	145	164	126	204	117	104	120	82	117	130	121	138	336
**NN**	179	339	179	447	262	144	1658	182	44	171	139	152	124	81	92	127	201	127	210	220	227	214	169	195	192	145	357	121	116	154	111	139	153	147	44	411
**KM**	449	773	365	812	602	203	351	2093	91	279	240	252	214	129	135	215	460	247	593	541	484	373	285	396	437	214	484	120	154	218	142	239	227	216	48	1046
**LS**	211	307	133	234	236	113	116	132	1123	139	117	122	113	77	105	185	224	102	371	233	198	155	117	135	175	127	204	68	80	129	96	117	120	119	24	509
**HB**	201	454	183	352	305	146	163	166	54	2364	404	347	216	96	117	149	247	133	232	272	249	199	174	227	393	165	284	96	122	213	145	275	169	155	62	884
**CC**	139	257	151	208	187	114	121	123	23	264	1441	217	151	64	78	107	160	113	154	176	163	146	127	154	242	123	178	51	78	144	106	181	128	120	21	427
**SY**	181	277	103	204	206	83	86	102	0	109	87	1020	83	47	75	155	194	125	341	203	168	125	87	105	145	97	174	38	50	99	66	87	90	89	51	479
**HH**	148	235	132	198	183	105	116	124	26	151	148	146	1721	75	120	130	193	118	167	182	168	143	125	142	217	118	168	47	67	134	93	132	124	115	16	494
**XN**	185	289	148	238	232	114	137	140	111	136	131	139	132	663	130	235	292	145	218	239	211	170	141	168	191	132	199	52	75	145	99	126	133	124	20	423
**YC**	165	264	136	214	209	103	122	129	45	141	127	137	193	110	795	204	279	135	190	221	189	149	129	155	187	124	180	49	76	138	92	126	126	119	12	406
**LZ**	202	333	170	286	259	136	154	156	102	163	143	157	157	193	178	1606	362	206	248	247	227	183	165	189	226	139	228	74	106	154	111	144	142	138	33	483
**XA**	351	743	267	537	453	164	208	227	117	243	206	239	243	178	195	355	3756	245	409	602	394	291	243	313	403	204	417	117	136	243	149	211	215	189	63	1065
**UM**	231	327	153	254	256	133	136	152	50	159	137	142	133	97	125	205	244	1330	391	253	218	175	137	155	195	147	224	88	100	149	116	137	140	139	44	529
**CD**	698	857	267	580	446	181	192	323	153	279	223	245	203	167	148	252	473	218	3907	423	507	336	230	277	383	262	527	129	131	257	154	242	234	207	74	1339
**ZZ**	263	643	212	415	350	152	152	191	91	210	164	171	147	98	110	150	301	152	480	2759	366	241	176	234	242	219	351	92	122	192	124	176	172	175	50	884
**WH**	285	766	236	563	387	163	169	216	72	218	172	195	143	102	113	151	297	140	562	413	2544	305	240	274	261	244	563	123	123	202	131	198	195	190	46	888
**CS**	184	409	176	456	258	134	152	168	30	160	127	147	118	94	85	118	214	121	402	234	343	1441	205	200	195	182	413	93	112	151	107	149	160	144	42	705
**NC**	183	376	226	352	335	131	160	152	42	154	138	148	130	78	92	131	194	123	206	224	281	233	1538	227	201	162	302	85	122	153	107	135	197	171	31	436

**HF**	178	621	189	284	416	121	147	141	55	154	141	153	125	82	100	129	213	129	201	279	299	203	194	2813	261	182	257	78	98	166	118	140	160	146	21	500
**TJ**	203	422	183	331	295	141	160	162	56	248	208	238	200	108	121	164	250	151	222	313	259	202	180	234	2626	155	280	96	118	209	138	202	163	150	40	1441
**NB**	173	549	215	293	1255	127	143	144	33	165	151	154	126	75	105	130	205	122	198	250	244	190	197	241	235	1474	244	80	117	168	120	152	165	152	26	489
**SZ**	326	666	354	1437	488	218	317	260	86	248	202	223	172	125	133	197	353	179	381	417	493	417	340	353	363	226	3350	162	232	212	143	193	337	262	86	906
**ZJ**	161	257	83	184	186	63	66	82	32	89	67	103	63	27	55	135	174	105	321	183	148	105	67	85	125	77	154	870	30	79	46	67	70	69	31	459
**ST**	123	221	153	568	175	108	123	107	11	108	97	102	73	28	35	80	133	76	140	145	149	140	129	126	143	119	353	95	801	110	60	98	168	135	17	234
**QD**	227	571	203	360	409	137	166	179	61	259	216	219	190	126	135	176	327	162	277	402	319	236	199	302	510	195	280	103	122	1874	277	206	175	157	42	1051
**YT**	151	247	73	174	176	53	56	72	22	79	57	93	53	17	45	125	164	95	311	173	138	95	57	75	115	67	144	56	20	69	980	57	60	59	21	449
**DL**	217	504	202	348	360	140	167	178	49	0	328	611	221	101	122	166	280	160	351	348	283	217	189	256	516	175	276	93	123	221	165	1549	169	156	38	931
**XM**	274	701	892	680	564	163	211	218	65	220	182	210	163	116	124	172	331	161	307	373	404	311	320	292	366	262	559	124	201	198	135	186	1860	558	66	853
**QZ**	154	316	522	261	251	113	141	136	21	137	126	135	108	56	75	108	169	113	173	196	197	163	167	167	184	145	229	68	123	145	109	129	384	1281	17	361
**SY**	285	466	219	433	370	779	206	213	76	234	186	202	166	122	124	159	308	170	341	328	279	247	188	227	307	174	313	125	127	170	127	165	179	169	342	768
**BJ**	383	873	337	781	621	214	294	315	112	408	323	365	361	155	177	271	524	252	478	700	510	397	323	442	958	254	599	142	176	338	198	302	272	257	86	5444

[a] *CQ: Chongqing; SH: Shanghai; FZ: Fuzhou; GZ: Guangzhou; HZ: Hangzhou; HK: Haikou; NN: Nanning; KM: Kunming; LS: Lhasa; HB: Harbin; CC: Changchun; SY: Shenyang; HH: Hohhot; XN: Xining; YC: Yinchuan; LZ: Lanzhou; XA: Xi'an; UM: Urumqi; CD: Chengdu; ZZ: Zhengzhou; WH: Wuhan; CS: Changsha; NC: Nanchang; HF: Hefei; TJ: Tianjin; NB: Ningbo; SZ: Shenzhen; ZJ: Zhanjiang; ST: Shantou; QD: Qingdao; YT: Yantai; DL: Dalian; XM: Xiamen; QZ: Quanzhou; SY: Sanya; BJ: Beijing*

[b] *A few of default values have been interpolated according to the values in other years.*
