# Supplementary material for: Urban networks among Chinese cities along "the Belt and Road": A case of web search activity in cyberspace
Source: PLoS One. 2017 Dec 4;12(12):e0188868. doi: 10.1371/journal.pone.0188868 (PMC5714330; doi:10.1371/journal.pone.0188868)
Supplement: S3 Table — (PDF) [file pone.0188868.s003.pdf]

S3 Table. Baidu index in 2016

	CQ	SH	FZ	GZ	HZ	HK	NN	KM	LS	HB	CC	SY	HH	XN	YC	LZ	XA	UM	CD	ZZ	WH	CS	NC	HF	TJ	NB	SZ	ZJ	ST	QD	YT	DL	XM	QZ	SY	BJ
**CQ**	4181	958	305	754	556	212	261	365	150	268	230	263	202	156	162	262	607	221	1189	482	625	388	286	348	421	304	637	158	171	289	165	228	284	293	100	1199
**SH**	475	5345	361	785	858	219	255	331	99	361	292	312	222	157	161	249	546	217	936	557	627	388	315	537	474	462	673	162	176	324	192	280	291	297	93	1448
**FZ**	180	417	1480	287	259	121	136	143	26	158	132	138	105	66	78	116	205	105	345	204	235	175	174	183	186	181	248	81	110	145	101	136	307	320	29	420
**GZ**	294	623	247	2742	353	209	264	232	60	226	191	200	150	114	110	168	293	155	569	310	423	330	233	243	279	223	806	298	200	199	130	178	217	214	83	803
**HZ**	310	1277	299	578	2765	176	203	235	68	283	232	242	180	116	124	190	753	166	656	413	466	307	263	415	370	738	472	129	145	266	155	233	245	241	70	1166
**HK**	192	303	143	283	210	827	150	164	26	158	137	135	115	72	77	125	184	111	268	198	203	169	133	145	172	148	234	124	91	129	86	122	132	129	150	401
**NN**	237	452	191	455	281	157	1292	195	46	194	155	167	136	93	95	140	242	129	726	242	279	219	167	202	221	185	386	129	117	170	119	154	163	172	61	545
**KM**	260	421	175	345	260	138	170	1422	77	176	151	159	126	126	94	145	246	125	441	240	260	203	157	188	209	172	282	104	112	161	114	148	155	156	51	611
**LS**	179	221	124	203	163	87	106	144	278	130	103	119	95	107	65	138	161	99	303	170	168	143	103	123	138	123	175	59	54	113	79	113	111	106	15	314
**HB**	250	596	204	418	345	166	167	193	63	3294	499	350	218	94	107	153	275	132	535	287	305	229	187	235	393	211	350	109	124	239	149	301	180	178	68	1073
**CC**	157	310	141	230	239	113	121	123	17	296	1208	216	145	59	69	109	171	102	259	183	187	151	125	148	125	138	200	72	83	146	103	181	128	128	25	481
**SY**	197	415	163	301	261	132	137	152	33	287	238	1357	164	81	96	132	202	122	588	209	235	182	149	170	234	180	261	88	113	167	121	441	151	148	39	635
**HH**	177	298	141	234	219	114	128	138	31	168	158	168	1581	83	113	137	210	125	267	201	201	157	133	149	229	136	203	70	79	145	108	144	132	130	24	571
**XN**	208	346	146	268	227	110	136	141	101	143	133	141	133	664	116	270	290	145	336	231	243	179	134	165	197	149	229	73	84	149	97	134	133	129	24	501
**YC**	198	340	140	237	210	106	118	135	40	159	131	138	178	104	765	207	278	127	298	203	213	163	127	154	189	139	203	67	81	144	93	130	130	124	18	462
**LZ**	235	388	168	303	242	128	147	164	83	172	150	153	145	209	157	1200	331	180	400	240	252	194	153	184	223	164	252	105	102	160	109	146	145	147	32	545
**XA**	401	839	262	556	434	168	194	245	104	270	232	244	226	171	180	327	2347	210	700	560	450	294	221	300	404	252	454	128	134	262	151	230	216	209	66	1172
**UM**	208	332	145	256	248	124	131	144	59	147	136	140	130	110	115	212	247	1410	349	224	219	171	134	161	191	144	220	84	94	147	105	130	137	134	30	482
**CD**	711	839	267	581	444	183	194	322	153	280	222	248	203	164	144	253	458	214	3740	414	507	328	230	281	373	263	526	132	132	253	153	239	231	209	74	1305
**ZZ**	249	643	202	390	337	144	158	181	80	200	174	177	147	107	102	151	306	150	416	2647	343	227	174	245	287	215	344	100	120	190	128	170	169	175	49	815
**WH**	329	873	256	612	429	165	194	220	72	229	200	208	157	106	115	174	364	158	627	494	2820	362	284	349	315	257	610	128	135	230	139	197	213	211	62	986
**CS**	227	496	216	545	309	155	206	180	55	179	154	171	134	100	99	150	243	139	300	290	449	1891	309	232	232	195	471	118	130	178	124	163	183	173	63	615
**NC**	253	553	239	427	375	151	180	184	53	199	165	188	149	94	94	147	242	134	319	274	358	281	1723	256	248	218	377	111	142	197	131	165	222	212	63	587

**HF**	216	889	189	324	363	125	147	155	61	181	243	168	126	88	90	140	255	129	771	272	323	218	187	2389	240	229	297	91	104	190	122	168	162	160	31	658
**TJ**	257	518	194	362	319	149	157	178	67	282	242	284	206	110	117	177	278	154	840	315	303	262	171	233	2447	194	312	100	117	236	148	225	169	167	57	1546
**NB**	215	755	211	351	1207	140	157	162	34	199	168	187	134	85	100	150	276	132	288	284	308	222	211	271	257	1730	294	102	128	217	139	180	193	187	45	600
**SZ**	444	1049	350	1546	567	250	342	303	101	308	241	279	197	144	145	237	428	196	619	493	691	496	410	373	399	307	4319	227	284	282	168	243	360	315	110	1224
**ZJ**	149	247	110	583	151	125	170	118	11	110	100	104	110	43	49	82	140	80	234	139	172	148	106	117	136	111	336	535	96	106	80	101	110	110	43	292
**ST**	142	263	152	583	171	104	116	107	9	111	102	113	111	32	41	78	136	74	203	144	167	137	118	123	145	133	339	110	609	115	76	106	159	152	17	274
**QD**	309	821	221	433	458	152	185	205	72	309	253	274	212	130	147	433	378	174	418	460	409	271	229	332	505	259	350	121	135	2202	298	245	204	190	61	1212
**YT**	137	253	109	169	148	86	93	102	13	143	113	119	100	49	44	100	142	82	255	154	145	121	89	114	149	118	151	47	45	278	636	125	109	94	12	387
**DL**	251	629	204	375	350	141	156	194	51	376	342	393	195	95	113	161	294	149	847	320	326	238	175	240	381	215	318	102	122	231	160	1362	177	173	51	993
**XM**	327	899	715	671	558	178	218	241	76	267	212	260	182	123	131	671	373	169	453	415	473	336	342	337	414	308	562	144	215	254	154	220	2012	849	87	990
**QZ**	182	417	363	303	261	119	137	145	26	164	139	148	106	67	75	120	208	108	394	213	233	179	159	175	201	181	264	84	122	160	111	143	410	1184	28	451
**SY**	361	608	222	483	404	787	220	243	90	277	218	243	184	128	125	483	339	177	476	371	349	285	223	259	333	217	354	141	138	212	141	194	198	196	493	867
**BJ**	449	1004	304	698	523	218	241	306	103	436	373	341	314	158	172	254	495	226	989	594	546	367	259	382	741	295	597	154	165	334	196	300	257	270	86	4424

[a] *CQ: Chongqing; SH: Shanghai; FZ: Fuzhou; GZ: Guangzhou; HZ: Hangzhou; HK: Haikou; NN: Nanning; KM: Kunming; LS: Lhasa; HB: Harbin; CC: Changchun; SY: Shenyang; HH: Hohhot; XN: Xining; YC: Yinchuan; LZ: Lanzhou; XA: Xi'an; UM: Urumqi; CD: Chengdu; ZZ: Zhengzhou; WH: Wuhan; CS: Changsha; NC: Nanchang; HF: Hefei; TJ: Tianjin; NB: Ningbo; SZ: Shenzhen; ZJ: Zhanjiang; ST: Shantou; QD: Qingdao; YT: Yantai; DL: Dalian; XM: Xiamen; QZ: Quanzhou; SY: Sanya; BJ: Beijing*

[b] *A few of default values have been interpolated according to the values in other years.*
